# Recovery of motor function of chronic spinal cord injury by extracellular pyruvate kinase isoform M2 and the underlying mechanism

**DOI:** 10.1038/s41598-020-76629-7

**Published:** 2020-11-10

**Authors:** Takahiro Kikuchi, Chihiro Tohda, Masato Suyama

**Affiliations:** Section of Neuromedical Science, Division of Bioscience, Institute of Natural Medicine, University of Toyama, 2630 Sugitani, Toyama, 930-0194 Japan

**Keywords:** Spinal cord diseases, Molecular neuroscience

## Abstract

In our previous study, we found that pyruvate kinase isoform M2 (PKM2) was secreted from the skeletal muscle and extended axons in the cultured neuron. Indirect evidence suggested that secreted PKM2 might relate to the recovery of motor function in spinal cord injured (SCI) mice. However, in vivo direct evidence has not been obtained, showing that extracellular PKM2 improved axonal density and motor function in SCI mice. In addition, the signal pathway of extracellular PKM2 underlying the increase in axons remained unknown. Therefore, this study aimed to identify a target molecule of extracellular PKM2 in neurons and investigate the critical involvement of extracellular PKM2 in functional recovery in the chronic phase of SCI. Recombinant PKM2 infusion to the lateral ventricle recovered motor function in the chronic phase of SCI mice. The improvement of motor function was associated with axonal increase, at least of raphespinal tracts connecting to the motor neurons directly or indirectly. Target molecules of extracellular PKM2 in neurons were identified as valosin-containing protein (VCP) by the drug affinity responsive target stability method. ATPase activation of VCP mediated the PKM2-induced axonal increase and recovery of motor function in chronic SCI related to the increase in axonal density. It is a novel finding that axonal increase and motor recovery are mediated by extracellular PKM2-VCP-driven ATPase activity.

## Introduction

Chronic spinal cord injury (SCI) is difficult to cure, and effective therapeutic approaches for chronic SCI have not been established practically. We previously found that a natural medicine-derived compound, acteoside, improved motor function, an increase in the density of raphespinal tracts, and synaptogenesis terminating at motor neurons in SCI mice when injected intramuscularly at the chronic phase^1^. As a result of the acteoside injection, pyruvate kinase isoform M2 (PKM2) was secreted from the skeletal muscle and then transferred to the plasma and the brain^1^. Extracellularly applied PKM2 enhanced axonal extension in primary cultured cortical neurons^1^. These results indicated that the effects of acteoside on chronic SCI might be mediated by PKM2 secretion from the skeletal muscles. However, we did not demonstrate that functional recovery in the chronic phase of SCI is completely mediated by extracellular PKM2 action.

PKM2 is a multifarious molecule. As a canonical role of glycolytic enzyme, the tetramer form of PKM2 catalyses the last step within glycolysis, the dephosphorylation of phosphoenolpyruvate to pyruvate^2^. In the dimer or monomer form, PKM2 shows a variety of localisation such as nuclear, mitochondrial, and extracellular distributions^3^. Concerning extracellular PKM2, secretion of PKM2 from colon cancer enhanced cell migration via the PI3K/Akt and Wnt/β-catenin pathway^4^, and secreted PKM2 from neutrophils facilitated wound healing via angiogenesis^5^, were reported. High serum levels of PKM2 in cancer patients also suggest that blood-circulating PKM2 may relate to tumour growth and angiogenesis^6–8^. It is known that secretion of PKM2 is mediated by exosomal delivery^9^. The human data also indicated that exosomes containing PKM2 were increased in blood by exercise^10^. However, the function of extracellular PKM2 in the central nervous system has not reported except for our study^1^.

In our previous study, we proposed that PKM2 is a new myokine, contributing to axonal extension and functional motor recovery in SCI mice^1^. However, the physiological role of extracellular PKM2 in the nervous system has never been studied, except by us, indicating no research findings of the signal pathway of extracellular PKM2 underlying the increase in axons. Therefore, this study aimed to identify a target molecule of extracellular PKM2 in neurons and investigate critical involvement of extracellular PKM2 in functional recovery in the chronic phase of SCI.

## Materials and methods

All experiments were performed following the Guidelines for the Care and Use of Laboratory Animals of the Sugitani Campus of the University of Toyama. All protocols were approved by the Committee for Animal Care and Use of the Sugitani Campus of the University of Toyama. The approval number for animal experiments is A2016INM-3 and A2019INM-3. All efforts were made to minimize the number of animals used.

### Materials

**Figure 1:**
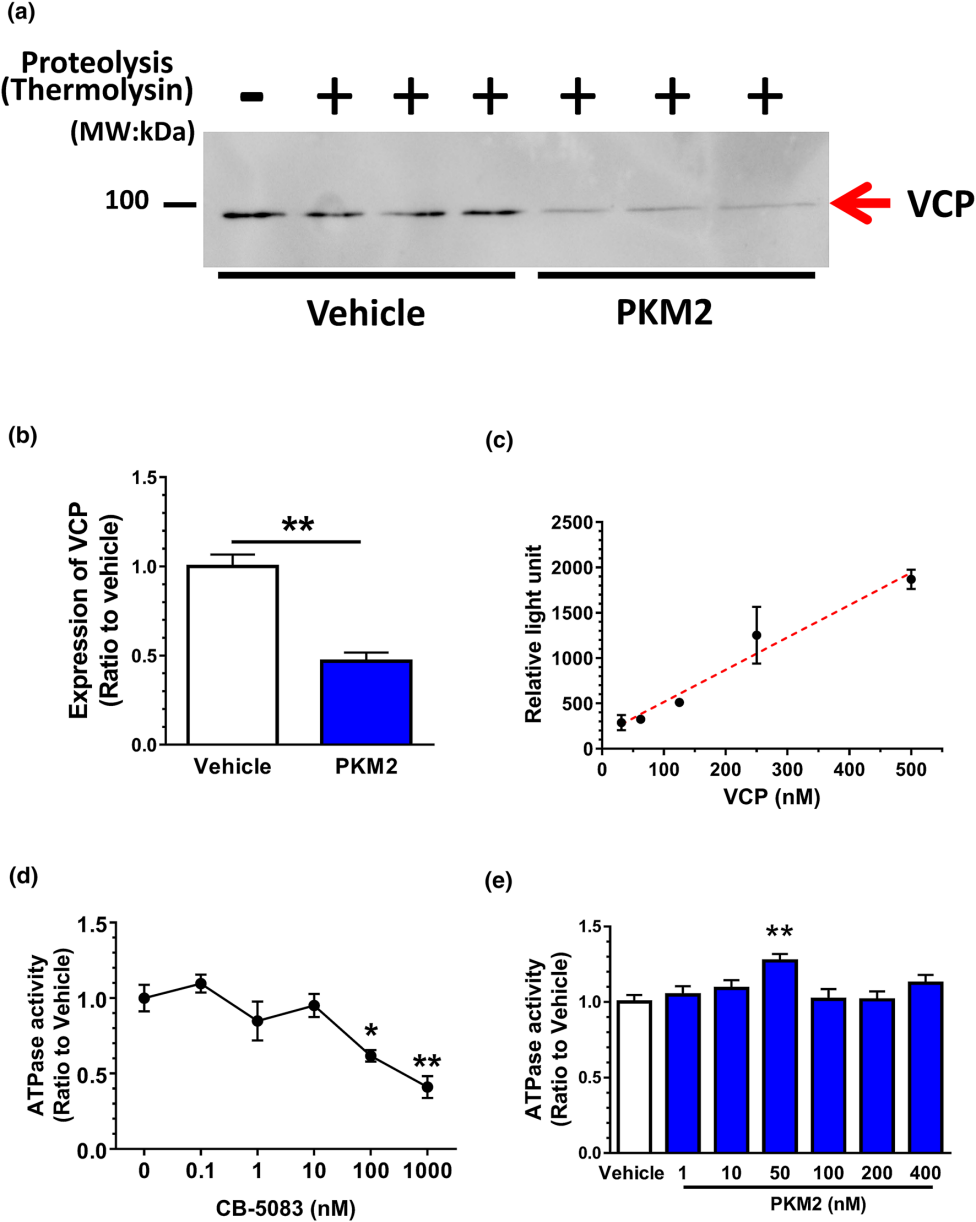
VCP is a target protein of extracellular PKM2. (**a**) Identification of VCP as a target of PKM2 by DARTS and western blot. Plasma membrane fraction of mouse cortical neurons were lysed, and recombinant PKM2 or vehicle solution was incubated with lysates for 30 min at room temperature. The mixture was proteolysed using thermolysin for 60 min at 37 °C and electrophoresed for western blotting for VCP. (**b**) Quantified band intensities of VCP. Student’s unpaired *t*-tests. **p < 0.01, n = 3. (**c**) A standard curve of VCP ATPase activity. (**d**) Inhibitory effect of CB-5083 on VCP ATPase activity. Used VCP concentration was 200 nM. One-way ANOVA with post hoc Dunnett’s test. *p < 0.05, **p < 0.01 vs. 0 nM, n = 3. (**e**) Effect of PKM2 on VCP ATPase activity. Used VCP concentration was 200 nM. **p < 0.01 vs. vehicle, n = 5–6.

**Figure 2:**
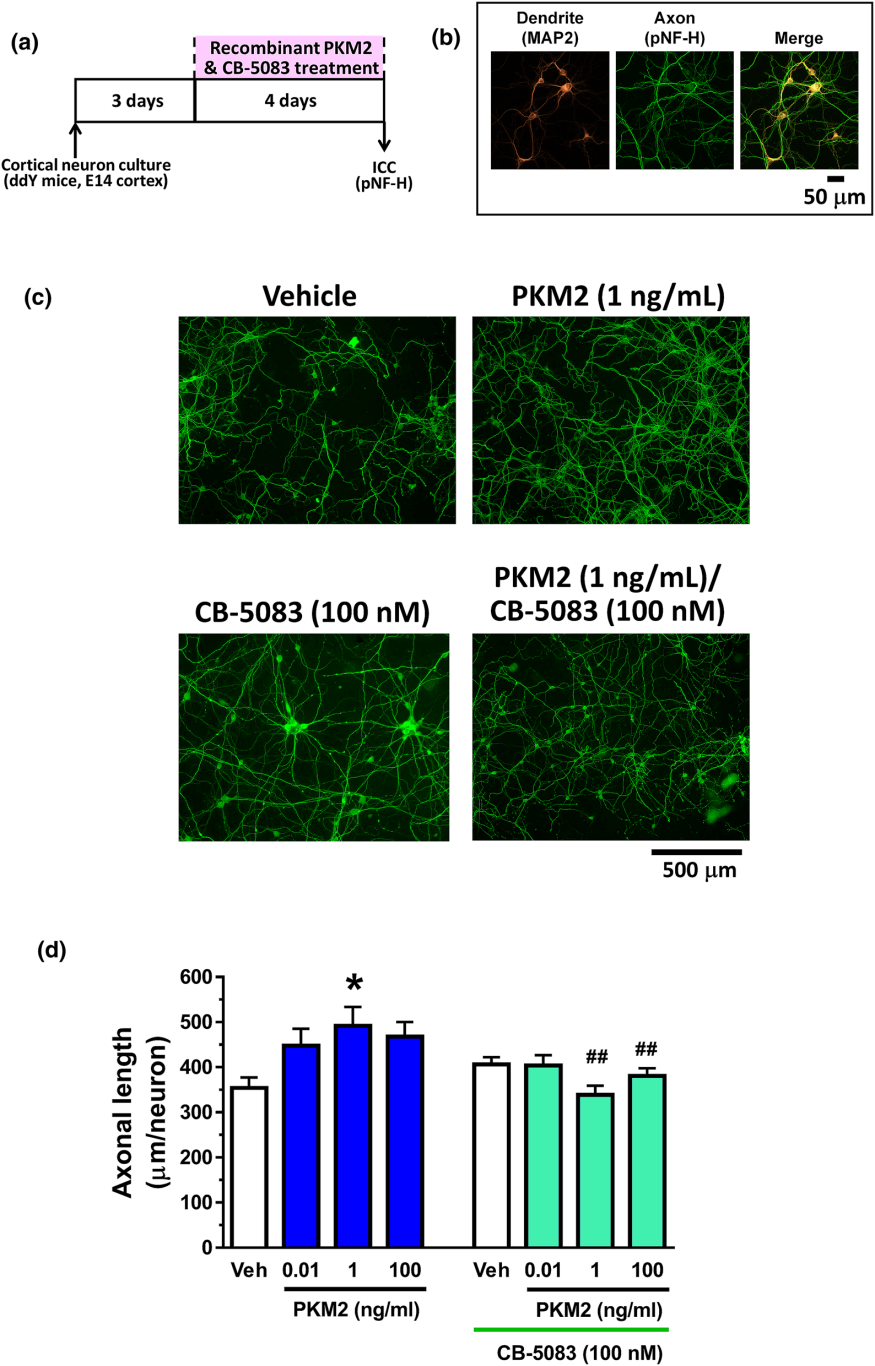
VCP ATPase activity is involved in PKM2-driven axonal extension. (**a**) Cortical neurons were cultured for three days and then treated with or without recombinant PKM2 and CB-5083. Four days after the treatment, the cells were fixed and double-immunostained for pNF-H and MAP2. (**b**) Representative images of MAP2-positive dendrite and cell body, pNF-H-positive axons and a merged image. Scale bar indicates 50 μm. (**c**) Representative photos of the immunostained axons are shown. Scale bar indicates 500 μm. (**d**) The density of pNF-H-positive axons was quantified for each treatment. *p < 0.05, one-way ANOVA with post hoc Dunnett’s test, ^**##**^p < 0.01 vs. same concentration of PKM2 without CB-5083 condition, two-way ANOVA, post hoc Tukey multiple comparison test. n = 9 − 11.

**Figure 3:**
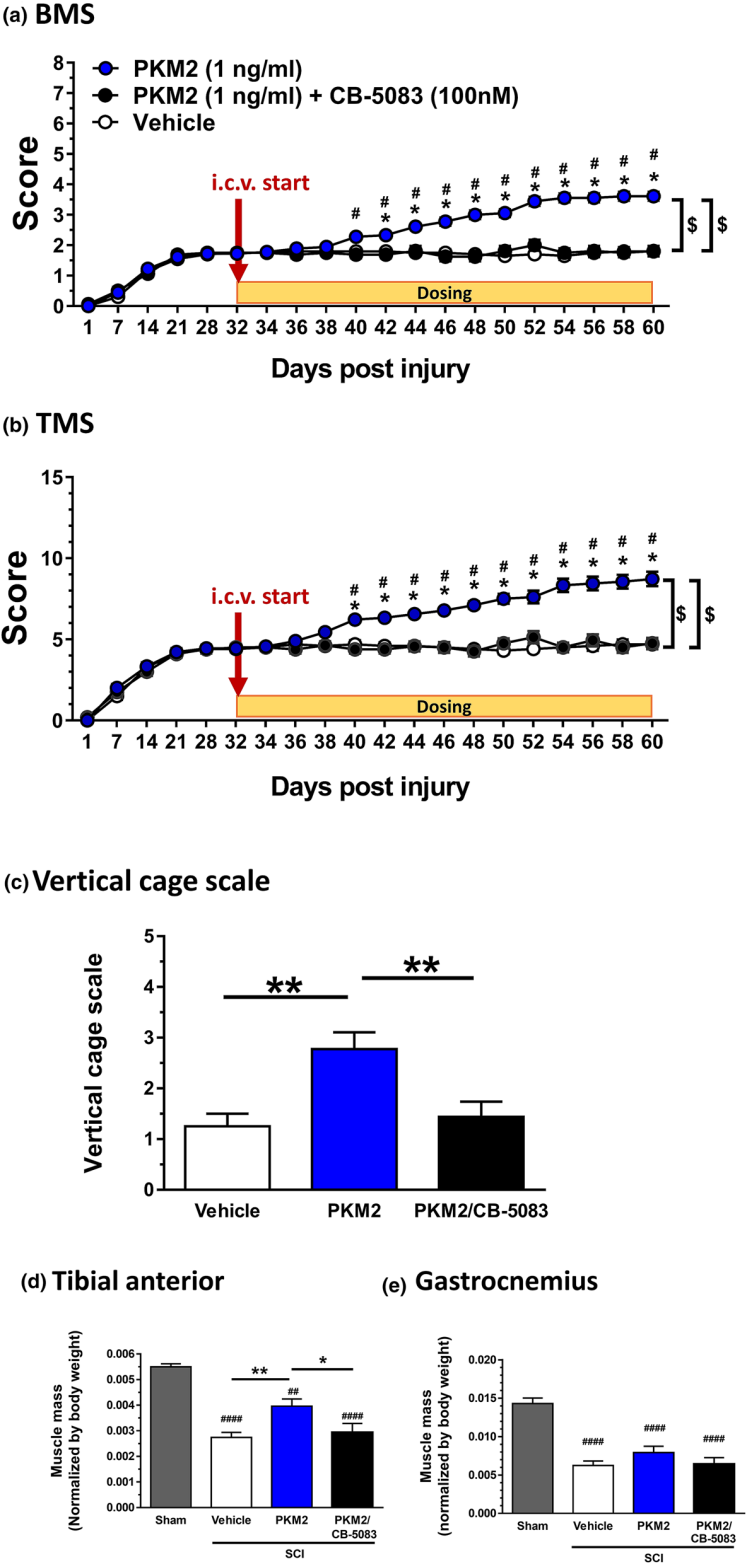
Effects of extracellular PKM2 i.c.v. infusion on locomotor function of mice with contusive spinal cord injury (SCI). Thirty-two days after SCI, recombinant PKM2 or vehicle solution was i.c.v. injected using a brain infusion cannula and an osmotic pump for 28 days. PKM2 was co-infused with or without CB-5083. Behavioural observations were performed during the pre-injection (32 days) and injection period (28 days). Hindlimb motor function of mice with SCI was evaluated using the Basso Mouse Scale (BMS; **a**), Toyama Mouse Score (TMS; **b**), and vertical cage scale (**c**). Vehicle group: n = 10 mice, n = 20 hindlimbs, PKM2 group: n = 9 mice; n = 18 hindlimbs, PKM2 + CB-5083 group: n = 8 mice; n = 16 hindlimbs. In (**a**,**b**), *p < 0.05, PKM2 vs. vehicle; ^**#**^p < 0.05, PKM2 vs. PKM-2 + CB-5083, repeated measures two-way analysis of variance (ANOVA), post hoc Bonferroni test in (**a**,**b**). ^**$**^p < 0.01, PKM2 vs. vehicle, PKM2 vs. PKM-2 + CB-5083; drug x day interaction by repeated measures two-way ANOVA in. In (**c**), **p < 0.01; one-way ANOVA, post hoc Tukey's multiple comparison test. Tibial anterior (**d**) and gastrocnemius (**e**) muscles were dissected to measure wet weights at the end of the behavioral observations. Muscle mass was normalized by the body weight at pre SCI operation. ^##^P < 0.01, ^####^P < 0.0001, vs. Sham, one-way ANOVA, post hoc Tukey's multiple comparison test. Sham group: n = 5, n = 10 hindlimbs, Vehicle group: n = 10 mice, n = 20 hindlimbs, PKM2 group: n = 9 mice; n = 18 hindlimbs, PKM2 + CB-5083 group: n = 8 mice; n = 16 hindlimbs.

**Figure 4:**
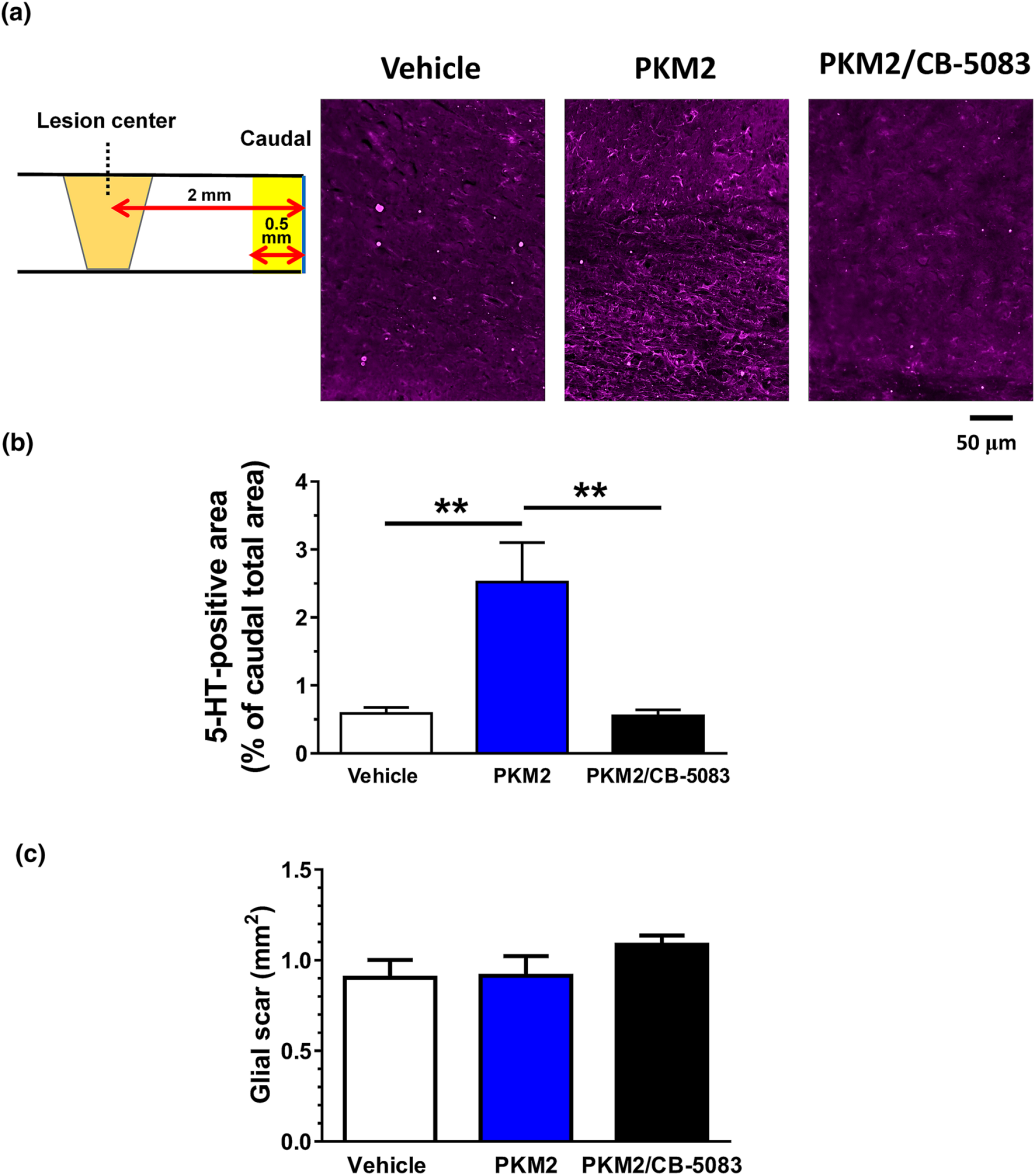
Effects of extracellular PKM2 i.c.v. infusion on increase in axons at the caudal site of lesion centre of mice with chronic spinal cord injury (SCI). PKM2 (final concentration in CSF; 1 ng/ml), CB-5083 (final concentration in CSF; 100 nM), or vehicle solution was mono or combined i.c.v. infused for 28 days, starting at day 32 after SCI. Fluorescent immunostaining was performed in spinal cord slices using the 5-hydroxytryptamine (5-HT) antibody. 5-HT–positive regions in the grey matter was quantified at caudal sites, 2 mm away from the lesion centre. (**a**) Representative images of 5-HT immunostaining at the caudal sites. In a scheme, yellow areas are quantified regions in the caudal site. Scale bar indicates 50 μm. (**b**) Quantified densities of 5-HT-positive axons are shown as percentages to measured caudal areas. (**c**) Quantified area of glial scar which is surrounded by GFAP-positive astrocytes. **p < 0.01, one-way ANOVA, post hoc Tukey's multiple comparison test. Vehicle group: n = 5 mice, PKM2 group: n = 5 mice, PKM2 + CB-5083 group: n = 5 mice.

**Figure 5:**
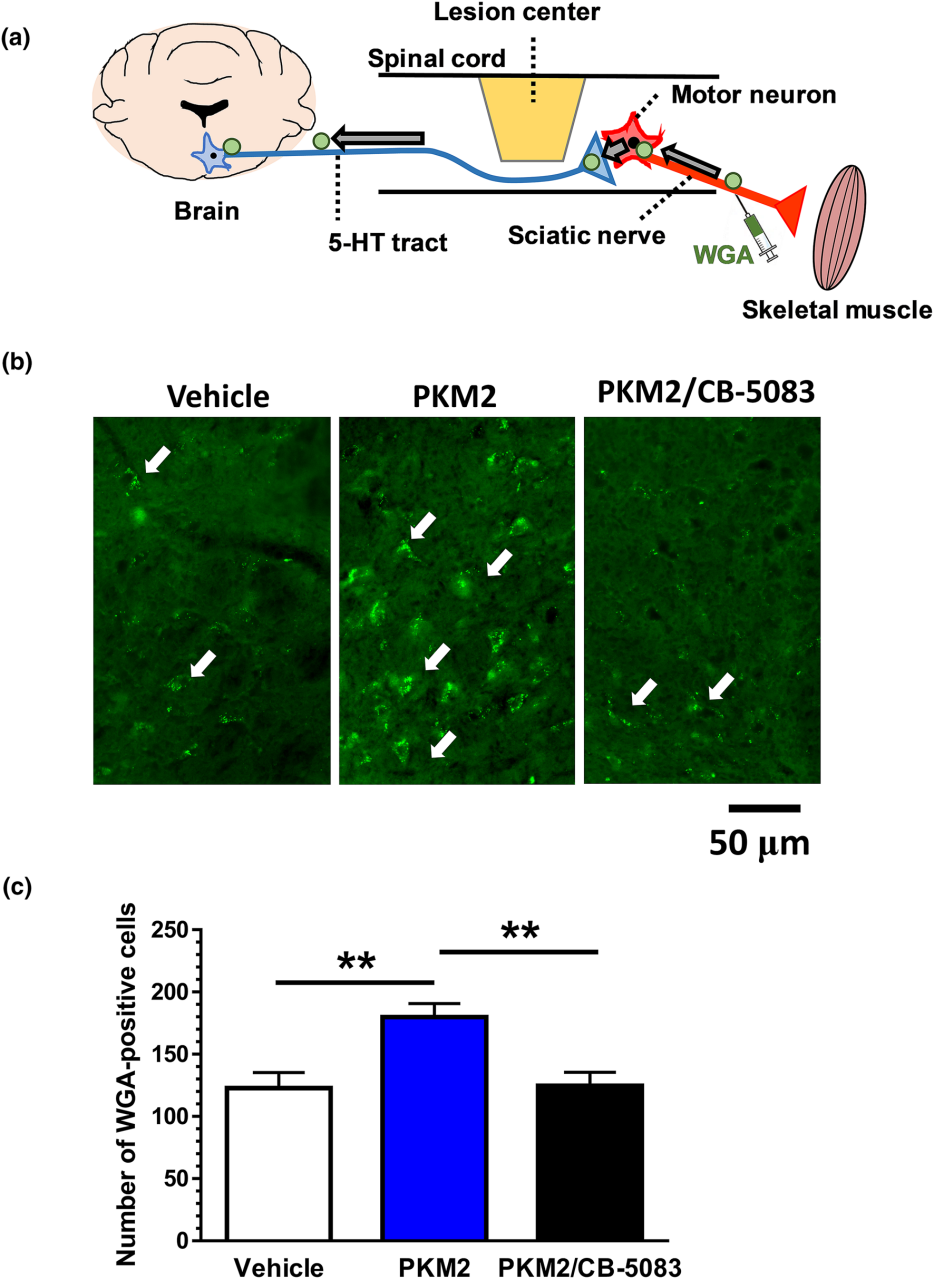
Increase in raphespinal neurons retrogradely labelled from sciatic nerve by PKM2 i.c.v. infusion. (**a**) Retrograde transsynaptic tracer WGA was injected in sciatic nerves of the left and right hindlimbs at 7 days before sacrifice. Labelled neurons were counted in raphe nucleus including the raphe magunus nucleus (RMg), raphe obscurus nucleus (Rob), and raphe pallidus nucleus (RPa). (**b**) Representative images of WGA-positive neurons in the raphe nucleus. Arrows indicate WGA-positive neurons. (**c**) Numbers of WGA-positive neurons. **p < 0.01, one-way ANOVA, post hoc Tukey's multiple comparison test. Vehicle group: n = 6 mice, PKM2 group: n = 6 mice, PKM2 + CB-5083 group: n = 7 mice. Scale bar indicates 50 μm.

Recombinant mouse PKM2 (Abcam, Cambridge, UK) was dissolved in sterile, distilled water (Figs. 1, 2, Supplementary Figs. 1–4) or artificial cerebrospinal fluid (ACSF) (Figs. 3, 4, 5, Supplementary Fig. 5, 6). Recombinant human valosin-containing protein (VCP) (Abnoba, Taipei, Taiwan) was dissolved in sterile, distilled water. VCP inhibitor, CB-5083 (Cayman, Ann Arbor, USA) was dissolved in DMSO at 1000 times concentration of final doses.

### Primary culture

The primary culture was performed as previously described^1^. Pregnant mouse and embryos of ddY mice (Japan SLC, Shizuoka, Japan) were anesthetized with isoflurane (FUJIFILM Wako Pure Chemical, Osaka, Japan). Embryos were removed from at 14.5 days of gestation. The cortices were dissected, and dura mater was removed. The tissues were minced, dissociated, and grown in cultures with neurobasal medium (Invitrogen, Grand Island, NY, USA) that included 12% B-27 supplement (Invitrogen), 0.6% d-glucose, and 2 mM l-glutamine in 8-well chamber slides (Falcon, Franklin Lakes, NJ, USA) coated with 5 μg/mL poly-d-lysine at 37 °C in a humidified incubator with 10% CO_2_. The seeding cell density was 2.2 × 10^4^ cells/cm^2^.

### The identification of putative PKM2 direct target protein in cultured neurons using drug affinity responsive target stability (DARTS) analysis

DARTS analysis was performed as described previously^11,12^. Cortical neurons were cultured for four days. The cell lysate was prepared using mammalian protein extraction reagent (M-PER) lysis buffer (Thermo Fisher Scientific, Waltham, USA) containing a protease and phosphatase inhibitor cocktail (Thermo Fisher Scientific). Cell lysate (2 μg protein) was incubated with 50 and 500 ng/ml recombinant mouse PKM2 for 30 min at 24 °C and each of these were proteolysed with 0.5 and 5 ng/mL thermolysin (Wako, Osaka, Japan), respectively, in a reaction buffer (50 mM Tris–HCl, pH 8.0; 50 mM NaCl; 10 mM CaCl_2_) for 30 min at 37 °C. To stop the reaction, 0.5 M ethylenediaminetetraacetic acid (pH 8.0) was added to each sample at a 1:10 ratio on ice. The samples were separated on 10% sodium dodecyl sulphate–polyacrylamide gel (SDS-PAGE). The proteins in the gels were visualized using a SilverQuest Kit (Invitrogen, Carlsbad, CA, United States). Four protein bands (indicated in Supplementary Fig. 1) were thinner in the sample treated with 500 ng/mL PKM2 compared to those of the sample treated with vehicle solution. The bands were excised from the gel, digested with trypsin, and analysed by mass spectrometry using a nano liquid chromatography-tandem mass spectrometry (LC–MS/MS) system (Japan Proteomics, Sendai, Japan).

### Binding assay by immunoprecipitation^11^

Recombinant mouse PKM2 (100 pmol) and recombinant human VCP (10 pmol) were coincubated for 1 h at 25 °C. Fifty µl of Dynabeads Protein G (Thermo Fisher Scientific) were treated with 1% bovine serum albumin for blocking in 0.01% Tween phosphate buffered saline for 30 min at 4 °C with rotation. Then, the protein G was incubated with a rabbit anti-PKM2 antibody (2 µL, Cell Signaling Technology, Danvers, MA, US) or normal mouse IgG (2 µL, Santa Cruz Biotechnology, Dallas, TX, US) for 30 min at 4° with rotation. Protein G and antibodies were cross linked by 50 mM dimethyl pimelimidate dihydrochloride (Tokyo Chemical Industry Co., Ltd., Tokyo, Japan) for 1 h at 25 °C with rotation. After washing protein G, incubated recombinant proteins were added, and the mixture was incubated for 2 h at 25 °C with rotation. For the elution of immunoprecipitants, the samples were mixed with LDS sample buffer and 0.1 M glycine–HCl (pH 2.8) for 5 min at 95° and then were loaded onto 10% polyacrylamide gels and electrophoresed. After electrophoresis, PKM2 or VCP in the gel were detected by Western blotting using a rabbit monoclonal anti-PKM2 antibody (1:1000, Cell Signaling) or a mouse monoclonal anti-VCP antibody (1:2000, Abcam) as first antibodies. Secondary antibodies against rabbit IgG (1:2000, Cell Signaling) and mouse IgG (1:2000, Abcam) were used.

### Western blotting

Western blotting was performed using samples after proteolysis in the DARTS analysis^11^. Lysates of the plasma membrane fraction of cultured neurons were prepared with the Mem-PER Plus Membrane Protein Extraction Kit (Thermo Fisher Scientific) after four days of in vitro culturing following the manufacturer’s protocol. Samples from the DARTS reaction were loaded onto a 10% SDS-PAGE. After electrophoresis, proteins in the gel were transferred to a nitrocellulose membrane (Bio-Rad, Berkeley, CA, United States) and blocked with 0.1% T-TBS containing 5% skim milk (Wako) at 24 °C. Subsequently, the membrane was incubated with a mouse monoclonal anti-VCP antibody (1:5000 for Fig. 1, Abcam) after blocking. The reaction was done in Can Get Signal solution 1 (Toyobo, Osaka, Japan) for 18 h at 4 °C. After washing, the membrane was incubated with horseradish peroxidase-conjugated secondary antibody against mouse IgG (1:2000, Abcam) in Can Get Signal solution 2 (Toyobo) for 2 h at 24 °C room temperature. Chemiluminescence of protein bands were detected by ECL Prime Western Blotting Detection Reagent (GE Healthcare) using an ImageQuant LAS 4000 system (GE Healthcare).

### ATPase activity assay

ATPase activity was measured by ADP-Glo Kinase Assay Kit (Promega, Madison, USA) following the manufacture’s manual. VCP and/or CB-5083 was incubated with ATP (20 μM) in kinase reaction buffer (40 mM Tris–HCl (pH 7.5), 20 mM MgCl_2_, 0.1 mg/mL bovine serum albumin) for 60 min at 37 °C. The solution stood for 40 min at room temperature after mixing with ADP-Glo reagent and further incubated with kinase detection reagent for 60 min at room temperature. Resultant luminescence was detected by Filter Max F5 plate reader (Molecular device, San Jose, USA).

### Measurement of axonal length

Evaluation of axonal length was performed as previously described^1^. For measurement of the density of axons, the cells were treated with or without recombinant mouse PKM2 or the vehicle solution (distilled water) for four days. The neurons were fixed with 4% paraformaldehyde for 90 min and immunostained with a polyclonal antibody against microtubule-associated protein 2 (MAP2, 1:2000, Abcam, Cambridge, UK) as a neuron marker. A monoclonal antibody against phosphorylated neurofilament-H (1:250, SMI-35, Covance, Dedham, MA, USA) was used as an axonal marker. Alexa Fluor 594-conjugated goat anti-rabbit IgG (1:600) and Alexa Fluor 488-conjugated goat anti-mouse IgG (1:600) were used as secondary antibodies (Molecular Probes, Eugene, OR, USA). Nuclear counterstaining was performed using DAPI (1 μg/mL, Sigma-Aldrich). The fluorescence images were captured with a 20 X objective lens using a fluorescence microscope system (Cell Observer, Carl Zeiss, Tokyo, Japan). Nine to thirty-three images (Fig. 2) were captured per treatment. The lengths of the pNF-H-positive axons were measured using a MetaMorph analyser (Molecular Devices, Sunnyvale, CA, USA), which automatically traces and measures the neurite length without measuring the cell bodies. Numbers of MAP2-positive and DAPI-positive cells were counted by the MetaMorph. The sum of the axon length was divided by the number of MAP2-positive neurons.

### SCI surgical operation and continuous administration of PKM2

Eight-week-old female ddY mice (SLC, Japan) were used for the SCI experiments. All mice were housed with access to food and water ad libitum and kept in a constant environment (22 ± 2 °C, 50 ± 5% humidity, 12 h light cycle starting at 07:00). The mice were anaesthetised with butorphanol tartrate (5 mg/kg, i.p., Meiji Seika Pharma Co., Ltd., Tokyo, Japan), medetomidine hydrochloride (0.75 mg/kg, i.p., Zenyaku Kogyo Co., Ltd., Tokyo, Japan) and midazolam (4 mg/kg, i.p., Fuji Pharma Co., Ltd., Tokyo, Japan). After laminectomy, contusion injury was given by dropping a 6.5-g weight from a height of 3.5 cm onto the exposed spinal cord at the level of T13 using a stereotaxic instrument (Narishige, Tokyo, Japan), as described previously^1^. During and after surgery, the mice were placed on a heating pad to maintain body temperature. Thirty-two days after SCI surgery, the mice were divided into three groups; vehicle solution (ACSF) (n = 10), 1 ng/mL PKM2 (n = 9), and 1 ng/mL PKM2 + 100 nM CB-5083 (n = 8). Mice were placed in a stereotaxic apparatus, and the head was kept in a fixed position. The scalp was shaved, followed by a sagittal midline incision to expose the skull. A cannula (Brain Infusion Kit 3, DURECT Corporation, CA, USA) was inserted to a lateral ventricle as follows: bregma − 0.22 mm, lateral to the left + 1 mm and − 2.5 mm depth. The free end of the cannula was connected to a micro-osmotic pump (Alzet model 1004) via a 3.5 cm piece of polyvinylchloride (PVC) tubing (Alzet). The cannula was fixed to the skull with Aron Alpha A “Sankyo” (Daiichi Sankyo, Tokyo, Japan). The pump was placed into a subcutaneous pocket in the back of the mouse. The infusion rate of the micro-osmotic pump was 0.11 ml/hr. As the vehicle solution, ACSF (containing 130 mM NaCl, 24 mM NaHCO_3_, 3.5 mM KCl, 1.3 mM NaH_2_PO_4_, 2 mM CaCl_2_, 2 mM MgCl_2_·6H_2_O, and 10 mM glucose at pH 7.4) was filled into the micro-osmotic pump and connected PVC tube. The micro-osmotic pump and tube were filled with 164 ng/mL of PKM2 and 16.4 mM CB-5083 that was dissolved in ACSF, considering that the pump efflux was 0.11 μL/hr and CSF was produced at a speed of 0.325 μL/min^13^. Thus, the final concentrations of PKM2 and CB-5083 were always approximately 1 ng/ml and 100 nM, respectively, when they were delivered to the CSF of the SCI mice. These doses are enough to induce axonal extension by PKM2, inhibiting the effect of PKM2 by CB-5083 (Fig. 2).

### Behavioural evaluation

For behavioural scoring after surgery, mice were placed in an open cage (black colour, 50.0 cm × 42.5 cm × 15.0 cm) and observed while moving free for three minutes. The motor function of the hindlimbs was evaluated using the Basso Mouse Scale (BMS)^14^, Toyama Mouse Score (TMS)^15^, and vertical cage test under 500-lx illumination. The vertical cage scale was established by us in this study. Wire netting (29.0 cm × 61.8 cm, grid width; 1.4 cm × 1.4 cm) was placed at a 70° angle. The mouse was put at the bottom of the net, and the climbing performance was observed and evaluated with appropriate scales independently for the left and right hindlimbs (Supplementary Table 1). Movements of the left and right hindlimbs were evaluated independently. Behavioural observations were performed once every seven days during pre-injection (32 days) and once every two days during the injection period (28 days).

### The wet weight of muscles and immunohistochemistry for 5-hydroxytryptamine (5-HT)-positive axonal tracts^1^

After the behavioural observations, the tissues of mice were isolated with perfusion by 4% paraformaldehyde in phosphate-buffered saline under anaesthesia by administration of a mix of three anaesthetics by i.p. injection. The tibial anterior and gastrocnemius were excised. The wet muscle mass was weighed using an electronic analytical balance with a precision of 0.1 mg. The muscle weight was normalised by body weight ay pre-SCI operation. The spinal cords were dissected at the T10-L1 level, soaked in 30% sucrose, embedded with cryomold 3 (Sakura Finetech Japan, Tokyo, Japan), and stored at − 30 °C until use. Sagittal sections of spinal cords were cut into 12 μm using a cryostat (CM 3050S; Leica Microsystems, Wetzlar, Germany). After post-fixed in 4% paraformaldehyde, the sections were immunostained for 24 h at 4 °C with the rabbit polyclonal anti-serotonin (5-HT; 1:500, ImmunoStar, Hudson, USA) and mouse monoclonal anti-glial fibrillary acidic protein (GFAP; 1:1000, Sigma-Aldrich) antibodies. Alexa Fluor 647-conjugated goat anti-rabbit IgG antibody (1:400, Life Technologies) and Alexa Fluor 594-conjugated goat anti-mouse plus IgG_1_ antibody (1:800, Life Technologies) were used as secondary antibodies. Nucleus was stained by 1 μg/mL DAPI. Images were captured using a fluorescence microscope (BZ-X710, Keyence, Osaka, Japan) and quantified using Image J (National Institutes of Health). The injured area was defined as inside surrounded by the GFAP-positive area, where the glial scar formed. Quantification was done in the three most centre slides of serial sections. Areas of 5-HT-positive axons in the grey matter, located at caudal positions 2 mm away from the injury site, were quantified. Region of interest (ROI) was selected as a caudal total area, and 5-HT-positive area was detected in ROI. The percentage of 5-HT-positive area to ROI area was calculated.

### Retrograde transsynaptic tracing by wheat germ agglutinin (WGA)

Seven days before the end of behavioural observations, 10% WGA solution (Thermo Fisher Scientific) was injected into the sciatic nerves of the right and left hindlimbs (4 μL/site), under anesthetization. The whole brain was dissected and soaked in 30% sucrose and stored at − 30 °C until use. The brains were then cut into 20 μm coronal sections using a cryostat (CM 3050S; Leica Microsystems). The quantified area was Bregma − 4.84 to − 7.48 mm where covered raphe magunus nucleus (RMg), raphe obscurus nucleus (Rob), and raphe pallidus nucleus (RPa). The sections were post-fixed in 4% paraformaldehyde. Images were obtained using a fluorescence microscope (BZ-X710, Keyence), and all WGA-positive neurons were counted in RMg, Rob and RPa.

### Hematoxylin and eosin staining

Brain slices from Bregma − 1.34 to − 7.48 mm were stained using Hematoxylin and Eosin Stain Kit (ScyTek Laboratories, Logan UT, US). Bright field images were captured by BZ-X710 microscope (Keyence).

### Statistical analysis

Data were expressed as mean ± standard error. To determine the statistically significant differences, GraphPad Prism 7 (GraphPad Software, San Diego, CA, USA) was used to perform one-way analysis of variance (ANOVA) post hoc Dunnett's test (Figs. 1d,e, 2d), or Tukey’s multiple comparisons test (Figs. 3c–e, 4b,c, 5c, Supplementary Fig. 4), repeated measures two-way ANOVA post hoc Bonferroni’s test (Fig. 3a,b, Supplementary Fig. 5), and two-tailed unpaired *t*-test (Fig. 1b). Data are shown as mean ± standard error. The significance level was set at 5%.

## Results

### Identification of a target molecule of extracellular PKM2 in neurons

Using DARTS analysis, direct binding proteins of PKM2 in neurons were comprehensively explored. The concept of this method is based on change of the structural conformation of the protein when a ligand binds to a target protein. Modified structure of the protein by binding alters the resistance against proteolysis, and it becomes harder or easier to degrade the target protein^16^. Whole-cell neuron lysates were incubated with recombinant PKM2 or vehicle. After the proteolysis reaction using thermolysin, the lysates were electrophoresed and silver stained. Four bands were thinner on the gel in 500 ng/mL PKM2 treatment than the vehicle solution-treated (Supplementary Fig. 1). Among those candidates, bands a, b, and c showed no reproducibility; therefore, we focused on molecular weight around 90 kDa of protein. The band was analysed by nanoLC-MS/MS, and the results indicated with a high possibility that the band was valosin-containing protein (VCP) (Score: 368, coverage 26%). To confirm VCP as the target protein of PKM2, we performed DARTS followed by western blotting. Considering that extracellular PKM2 seems to not enter inside cells, we supposed binding proteins of extracellular PKM2 was possibly located on the plasma membrane. Therefore, plasma membrane lysates of the cortical neuron were used. Incubation with recombinant PKM2 facilitated degradation of VCP (Fig. 1a,b, Supplementary Fig. 1). Direct binding of PKM2 and VCP was confirmed by co-immunoprecipitation experiment (Supplementary Fig. 3). The mixture of recombinant PKM2 and recombinant VCP was immunoprecipitated by anti-PKM2 antibody or control normal mouse IgG. Precipitants were immunoblotted by anti-PKM2 and anti-VCP antibodies. Results indicate VCP is coprecipitated with PKM2, suggesting direct interaction of PKM2 and VCP.

VCP has many functions such as ER-associated protein degeneration^17^, cell division, organelle biogenesis, nuclear envelope formation, and protein degradation via the ubiquitin–proteasome system^18^. Since those varieties of cellular activity of VCP are associated with ATPase, ATPase activity was evaluated under the existence of recombinant VCP. In vitro ATPase activity was increased in a dependent manner of VCP concentration (Fig. 1c). CB-5083, a potent and selective VCP ATPase inhibitor, was incubated with 200 nM recombinant VCP. Doses of 100 nM and 1000 nM of CB-5083 significantly inhibited ATPase activity (Fig. 1d), indicating the detected ATPase activity is derived from VCP. Using this assay, an effect of PKM2 on the ATPase activity with 200 nM VCP was investigated. Recombinant PKM2 (50 nM) significantly elevated ATPase activity, suggesting that PKM2 stimulates VCP activity at that dose (Fig. 1e).

### PKM2-induced axonal extension was inhibited by VCP inhibitor

Recombinant PKM2 (1 ng/mL) treatment for four days significantly enhanced axonal length in cultured cortical neurons (Fig. 2c,d). Neurites were differentiated to axons and dendrites in this experimental condition (Fig. 2b). Simultaneous treatment with PKM2 (1 ng/mL) and CB-5083 (100 nM) did not increase the axonal length, indicating that extracellular PKM2-induced axonal extension is mediated by VCP. PKM2 and CB-5083 treatments gave no change of neuronal numbers (Supplementary Fig. 4). Dendrite lengths were not changed by PKM2 or CB-5083 treatment (data not shown).

### Extracellular PKM2-VCP signalling increases axonal density and improves motor function in chronic spinal cord-injured mice

We investigated the effect of extracellular PKM2-VCP signalling on functional recovery in the chronic phase of SCI mice. Thirty-two days after injury, continuous administration of recombinant PKM2 or vehicle solution, artificial cerebrospinal fluid (ACSF) into the lateral ventricle, was started using a micro-osmotic pump for 28 days. The concentrations of PKM2 and CB-5083 were maintained at approximately 1 ng/mL and 100 nM in the CSF, respectively, during the administration period, which are effective doses in culture cell experiments (Fig. 2d). The hindlimb motor functions were evaluated by Basso Mouse Scale BMS (Fig. 3a), TMS (Fig. 3b), and vertical cage scale (Fig. 3c). The scores of PKM2-treated mice gradually and significantly elevated compared to the scores of vehicle-treated mice eight days after intracerebroventricular (i.c.v.) infusion in BMS and TMS. In each scoring, two-way repeated measures ANOVA showed a significant difference for time × drug interactions among the vehicle-treated group and PKM2-treated group (F(19, 646) = 1.68, p < 0.05 in BMS; F(19, 646) = 1.685, p < 0.05 in TMS). During the 10–28 days post i.c.v. infusion, post hoc Bonferroni analysis indicated that both scores of the PKM2-treated group were significantly increased compared to those of the vehicle-treated group. In each scoring, two-way repeated measures ANOVA showed a significant difference for time × drug interactions among the PKM2-treated group and PKM2/CB-5083-treated group (F(19, 608) = 25.59, p < 0.0001 in BMS; F(19, 608) = 24.07, p < 0.0001 in TMS). During the 8–28 days post i.c.v. infusion, post hoc Bonferroni analysis indicated that both scores of the PKM2/CB-5083-treated group were significantly decreased compared to those of the PKM2-treated group.

At 27 days after i.c.v. infusion, the vertical cage test was performed. Scale point of PKM2-treated mice was significantly higher than that of vehicle-treated mice (Fig. 3c). However, simultaneous infusion of CB-5083 significantly reduced the score.

Wet weights of tibial anterior and gastrocnemius were significantly reduced in SCI mice compared with sham-operated mice (Fig. 3d,e). PKM2 i.c.v. infusion significantly enhanced the weight of the anterior tibial muscle and PKM2/CB-5083 i.c.v. co-infusion did not increase muscle weight. No significant changes in body weights were observed among the three groups during the experimental period (Supplementary Fig. 5).

After the behavioural tests, spinal cord and brain tissues were isolated. Slices of the spinal cord that included the lesion area were prepared and immunostained with the raphespinal tract marker 5-HT (Fig. 4a). The raphespinal tract is serotonergic and one of the major descending tracts that modulates the excitability of motor neurons. To decide the lesion area, the GFAP-positive glial scar area was stained. PKM2 i.c.v. infusion increased the density of 5-HT-positive axons at the caudal lesion site in mice with chronic SCI (Fig. 4a,b). CB-5083 infusion completely inhibited PKM2-induced increase in the density of axons. Areas of glial scar were not changed by PKM2 or PKM2/CB-5083 treatment (Fig. 4c).

A retrograde transsynaptic tracer, WGA, was injected in the sciatic nerves of the right and left hindlimbs. We counted WGA-positive neuronal cell bodies in the entire area of the raphe nucleus, including the RMg, Rob and RPa. The number of WGA-labelled neurons was significantly increased in PKM2-treated SCI mice compared to vehicle-treated SCI mice (Fig. 5b,c). While CB-5083 treatment significantly decreased the numbers of WGA-positive neurons in the raphe nucleus. Hematoxylin and eosin staining of brain slices indicates no obvious cell losses in the motor area and raphe nucleus area in three groups (Supplementary Fig. 6).

## Discussion

This study is the first report to indicate that extracellular PKM2 infusion to the lateral ventricle recovers motor function in the chronic phase of SCI mice. The improvement of motor function was associated with axonal increase, at least of raphespinal tracts connecting to motor neurons directly or indirectly. Since target molecules of extracellular PKM2 in neurons have not been determined, a comprehensive analysis was performed. VCP was identified as a direct target protein of PKM2, particularly its ATPase activity related to induction of axonal extension. The VCP-mediated axonal extension is a novel finding, although the downstream pathway of the upregulation of ATPase remains unknown. Additionally, the binding modes of PKM2 and VCP have not been identified yet. Various cofactors directly interacting with VCP have been identified by proteome analysis^19–21^. Although cofactors for VCP usually interact with VCP interaction motif^22^, ubiquitin regulatory X domain^23^, and VCP binding motif^24^, such domains are contained in the PKM2 sequence. Further study for identifying the binding modes of PKM2 and VCP in detail are expected.

Generally known VCP function covers a variety of phenomena, such as protein degradation of ER-related^25–27^, mitochondria-related^28,29^, and ribosome-related^30^, controlling autophagy^18^, molecular chaperone^31,32^ and so on. VCP function relating to axonal formation has hardly been investigated. Only one study indicated that VCP bound to the slow Wallerian degeneration protein (Wld^s^). However, VCP is not involved in the Wld^s^-mediated axon protection^33^.

VCP protein expresses in the entire region of the brain and spinal cord (the human protein atlas: https://www.proteinatlas.org/). VCP expression and PKM2 expression in the spinal cord of chronic (eight weeks post-injury) SCI mice are not decreased compared to that in an uninjured spinal cord^34^. As we previously found, extracellular PKM2 is secreted from skeletal muscle and transferred to the central nervous system by systemic circulation^1^. Although we detected an increase in the density of raphespinal tracts in this study, other descending tracts and interneurons might also be extended by extracellular PKM2 stimulation. Skeletal muscle atrophy is observed to be severe in SCI mice (Fig. 3d,e), and it partially recovered with PKM2 i.c.v. infusion. It is unlikely that PKM2 infused into lateral ventricle affected the hindlimb muscles. If anything, improved hindlimb movement might increase muscle mass.

In summary, extracellular PKM2 increases the density of axons and motor function when applied to the brain in the chronic phase of SCI mice. The increase in axons and motor recovery are mediated by extracellular PKM2-VCP-driven ATPase activity.

## Supplementary information

Supplementary Information

## Data Availability

All data needed to evaluate the conclusions in the paper are present in the paper or the Supplementary Materials.
