# Supplementary figures and images for: Recovery of motor function of chronic spinal cord injury by extracellular pyruvate kinase isoform M2 and the underlying mechanism
Source: Sci Rep. 2020 Nov 10;10:19475. doi: 10.1038/s41598-020-76629-7 (PMC7656253; doi:10.1038/s41598-020-76629-7)

## Slide 1
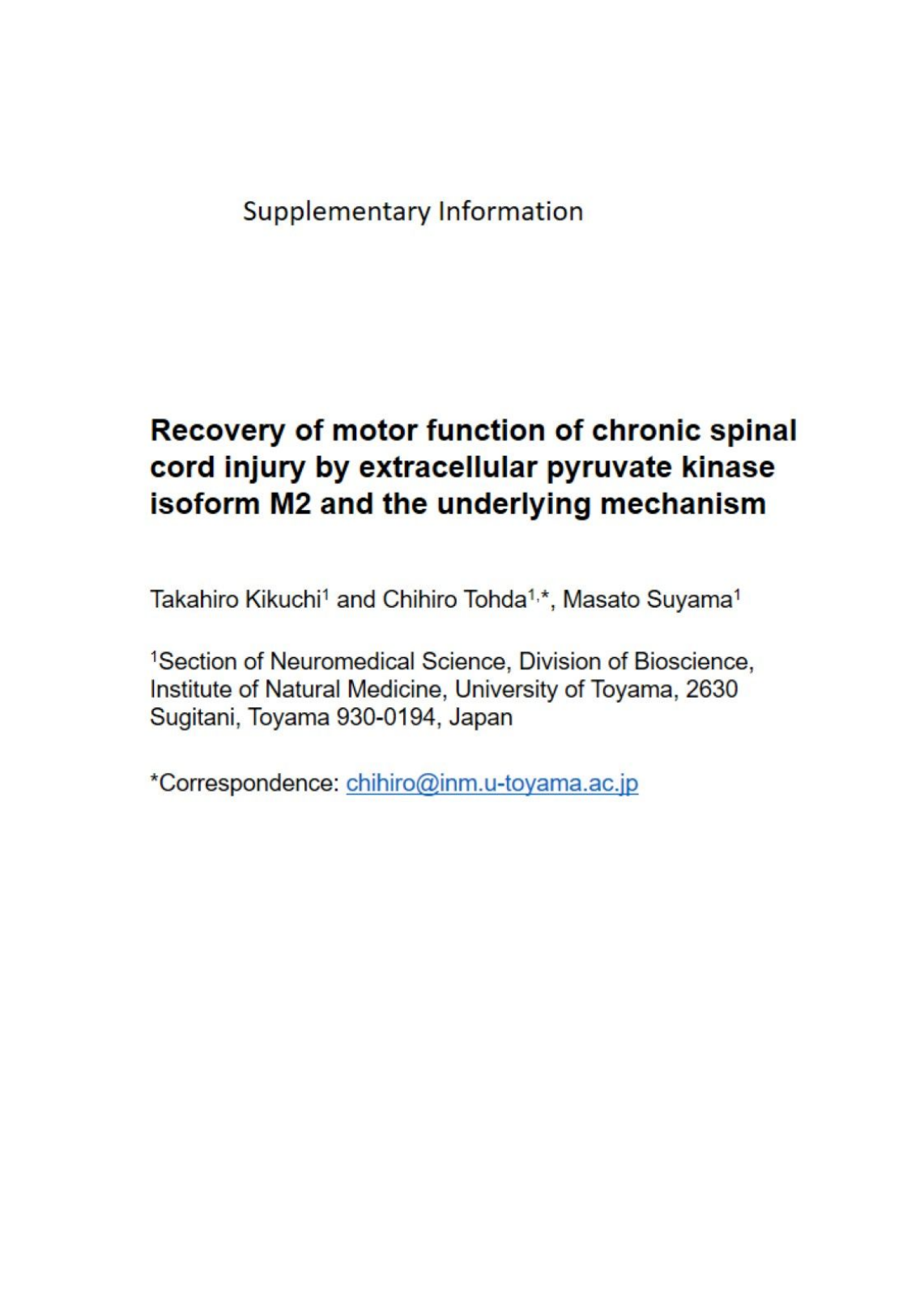

#

## Slide 2
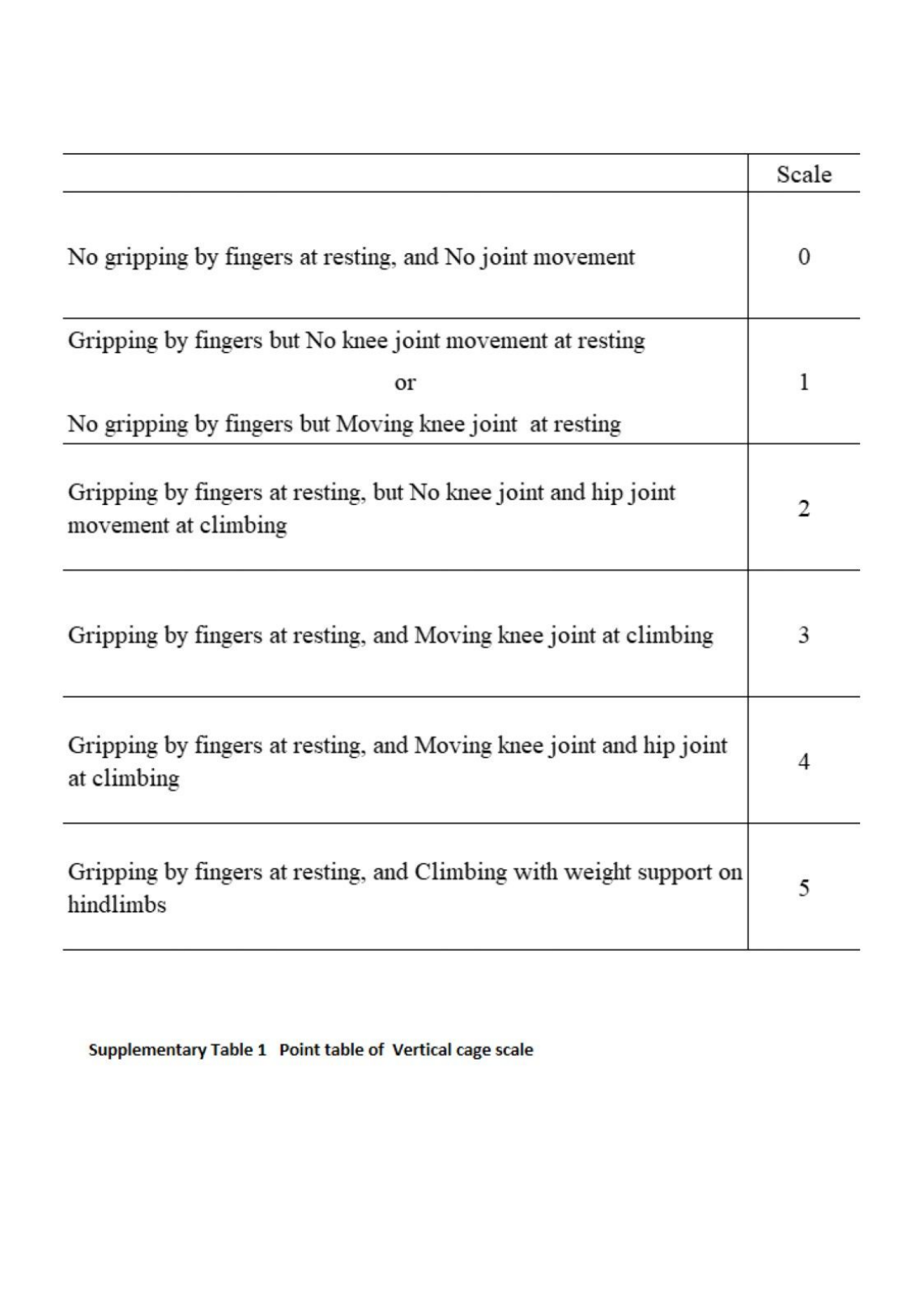

#

## Slide 3
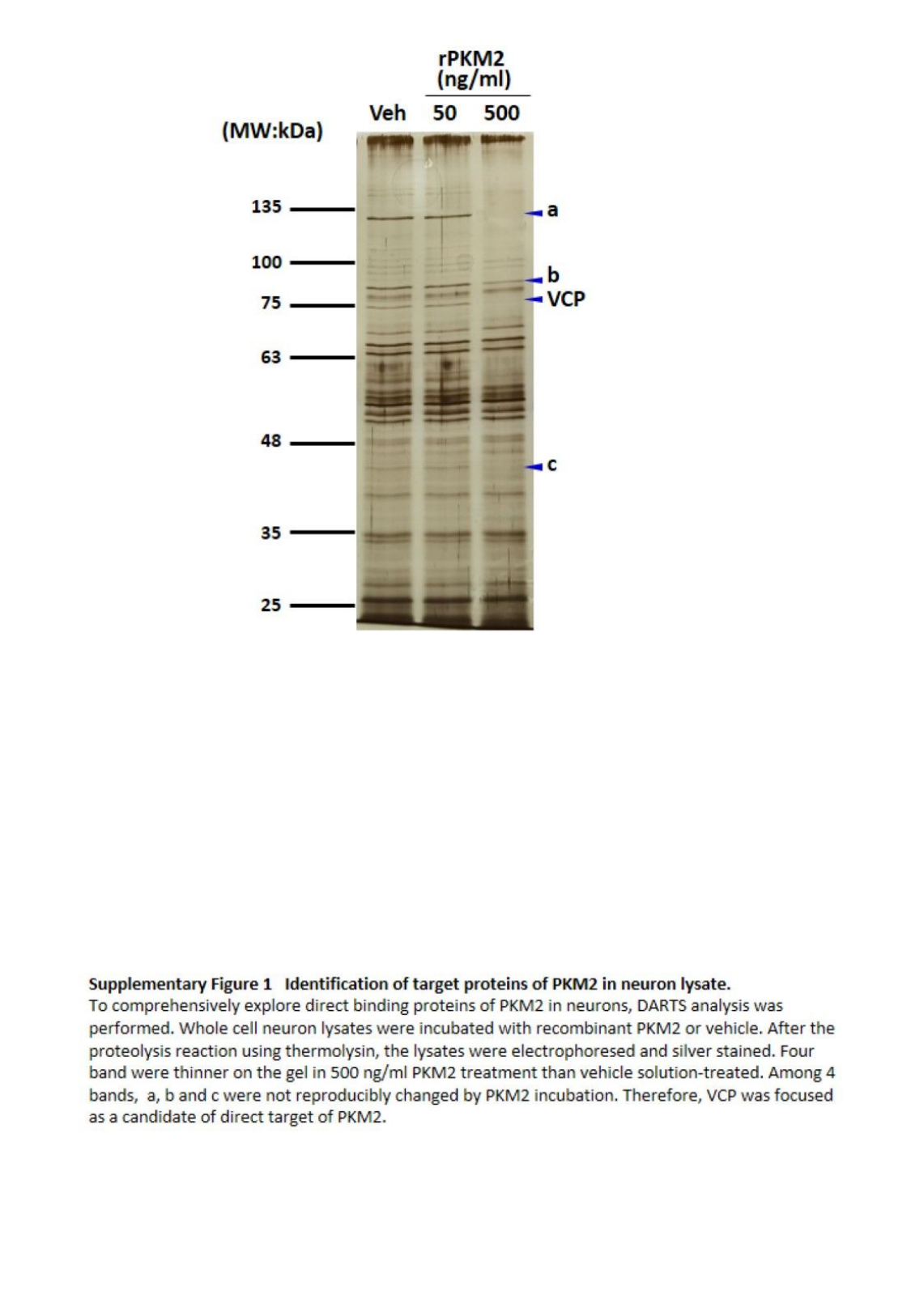

#

## Slide 4
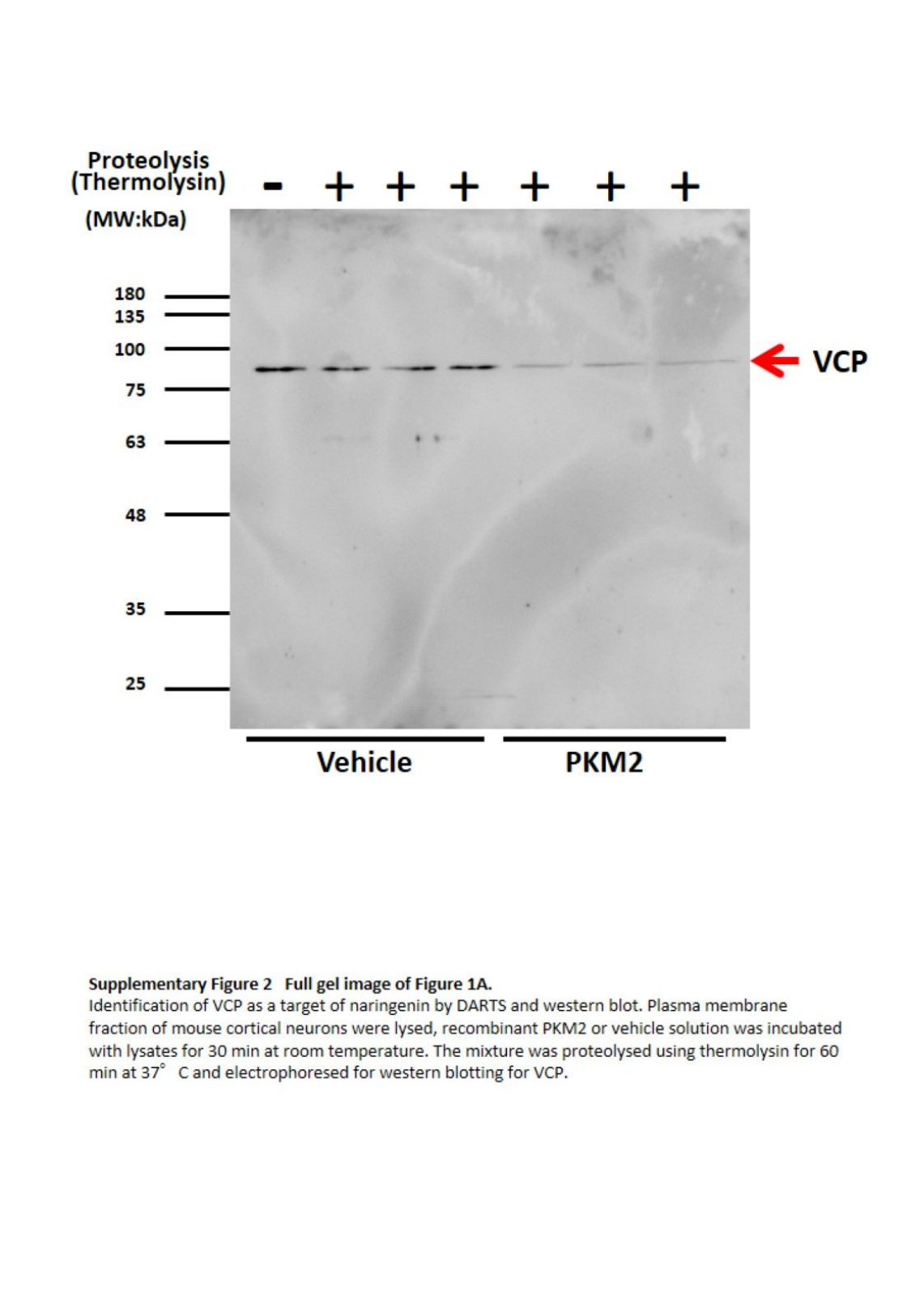

#

## Slide 5
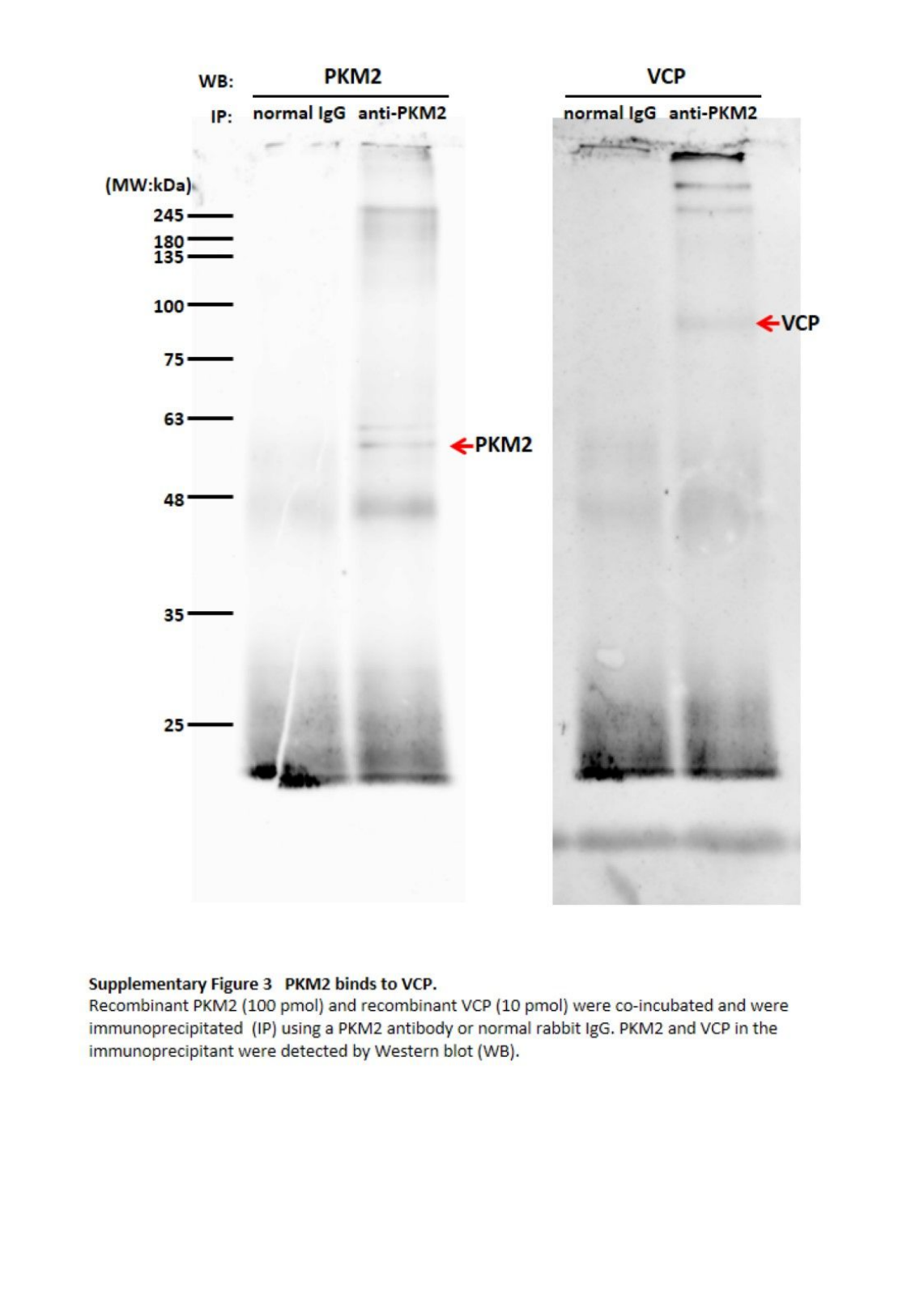

#

## Slide 6
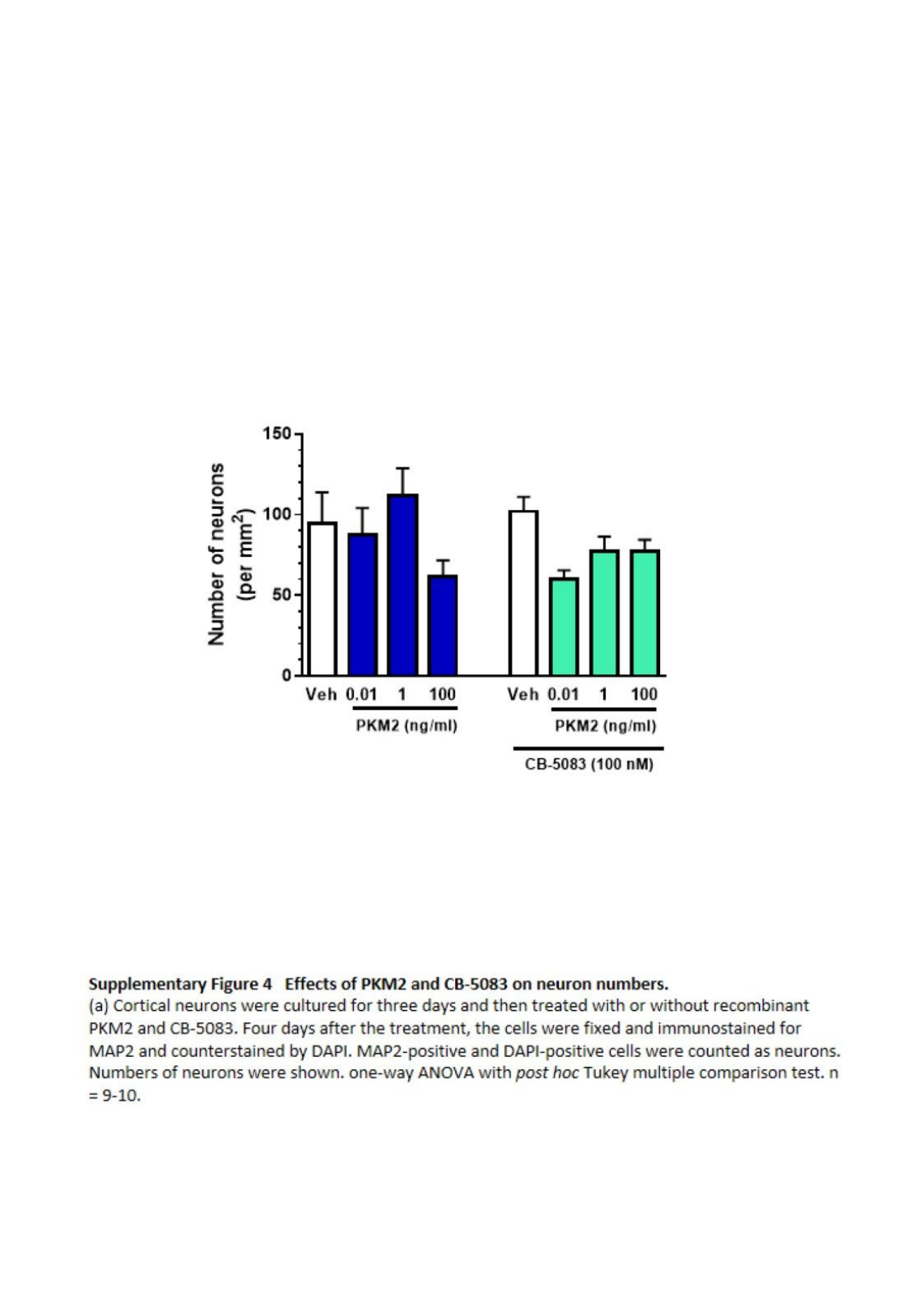

#

## Slide 7
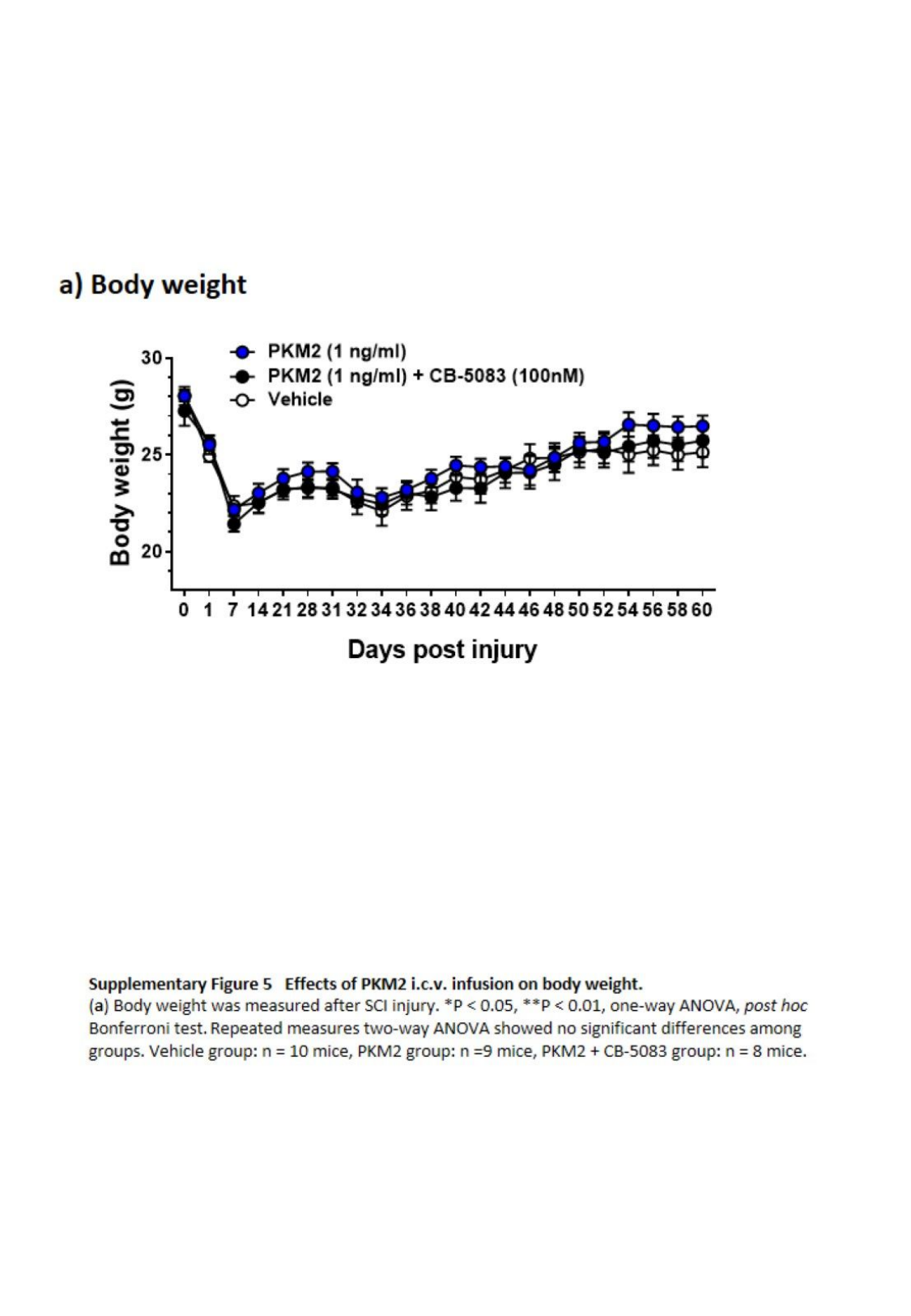

#

## Slide 8
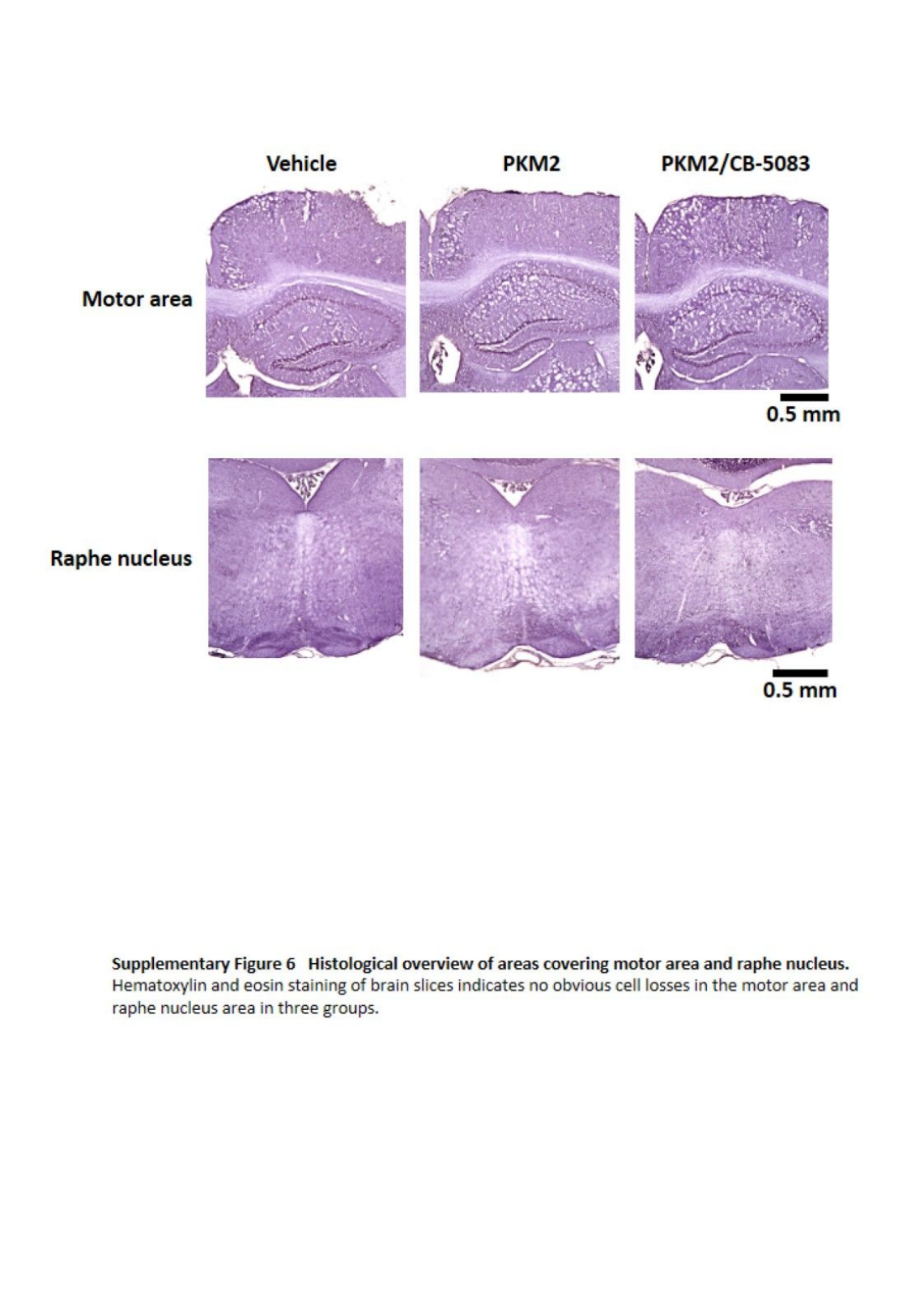

#

Supplement: Supplementary file 1 — Supplementary Information [file 41598_2020_76629_MOESM1_ESM.pptx]
